# Study on the Spontaneous Combustion Law of Coal Body around a Borehole Induced by Pre-extraction of Coalbed Methane

**DOI:** 10.1021/acsomega.4c02672

**Published:** 2024-09-12

**Authors:** Jun Guo, Xuanchi Zhang, Yin Liu, Guobin Cai, Hua Liu, Changming Chen, Lei Wang

**Affiliations:** † College of Safety Science and Engineering, Xi’an University of Science and Technology, Xi’an 710054, China; ‡ Key Laboratory of Western Mine and Hazard Prevention, Ministry of Education of China, Xi’an 710054, China; § Shaanxi Xiaobaodang Mining Co., Ltd., Yulin 719399, China

## Abstract

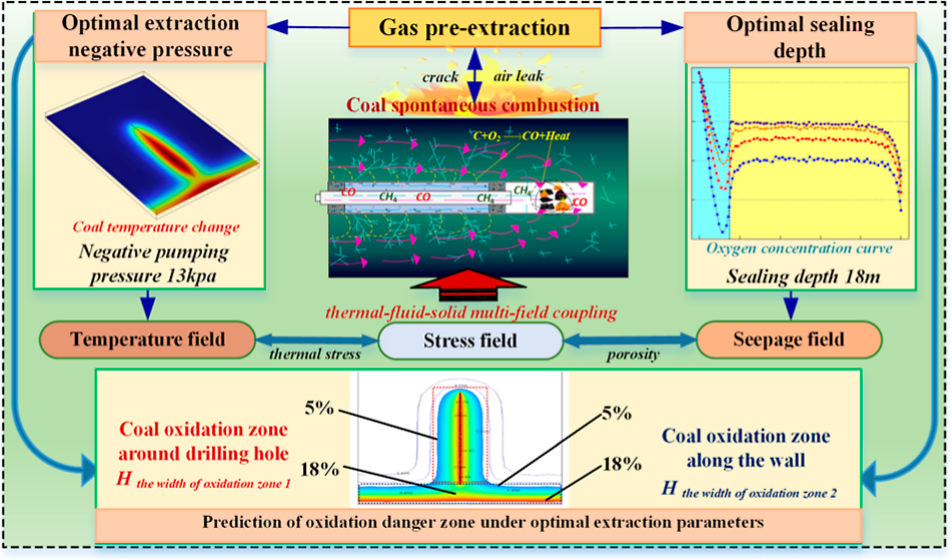

To address the challenges associated with the high gas content, high pressure, and low permeability coefficient in deep coal seams, strategies such as infilling boreholes and increasing the negative pressure of extraction are commonly implemented to alleviate issues related to coalbed methane extraction. However, long-term mining pressure can lead to the development of cracks in the coal seam near the borehole, thereby creating air leakage channels, which could potentially impact the oxygen supply during the extraction process. This leads to secondary disasters such as the spontaneous combustion of coal and gas explosions, considerably impacting the life and health of underground workers. To solve this issue, a thermal–fluid–solid coupling model for the working surface was constructed based on numerical simulation software, taking into account the multimechanism coupling effect of coal seam gas. The laws of coal oxidation and spontaneous combustion induced by coalbed methane extraction around boreholes were studied. The variation laws of the oxygen concentration, coal temperature, and oxidation heating zone around the borehole under different extraction conditions were simulated and analyzed. The findings demonstrate that the negative extraction pressure enables the gas to penetrate the fracture zone of the borehole, leading to an increase in the oxygen consumption rate and coal temperature around the borehole with an increase in negative extraction pressure. The coal gas leakage surrounding the borehole reduces as the sealing depth increases, and both the heating rate of coal and oxygen volume fraction show a downward trend. The fitting relationship between the negative pressure of drainage, depth of sealing, and temperature change in the coal body surrounding the boreholes was identified. It was determined that the negative pressure of 13 kPa for borehole drainage and a sealing depth >18 m are the optimal extraction parameters. The range of the oxidation zone and the position of the boundary line under this parameter were predicted, and the position function of the dangerous area of oxidation heating was defined. The research results have remarkable implications for the coordinated prevention and control of gas and coal spontaneous combustion in coalbed methane predrainage boreholes, as well as for efficient prevention and control of CO in on-site gas extraction boreholes, thus ensuring efficient and safe gas extraction.

## 1 Introduction

China is one of the world’s largest coal producing countries, rich in coal resources. In recent years, the mining of shallow coal resources has gradually depleted, leading the coal industry to transition to deep mining.^1^ The efficiency of gas predrainage in China’s coal seams is substantially impacted by their low pressure, low permeability, low saturation, and high adsorption characteristics, resulting in an increase in the occurrence of serious mine gas disasters.^2^ Several domestic mines often implement strategies such as encryption drilling and prolonging the drainage time to alleviate the issue. However, during the extraction, cracks form in the rock surrounding the roadway and borehole because of the stress of the coal seam and long-term mining pressure.^3,4^ Difficulties in precisely controlling extraction parameters and sealing boreholes can lead to significant air leakage, which provides a continuous supply of oxygen to coal bodies. This promotes spontaneous combustion and can lead to secondary disasters such as gas explosions.^5−7^ These issues compromise the safety of coalbed methane mining and pose serious risks to the health and safety of underground workers.^8−10^ Consequently, the key to effectively addressing the issues related to coalbed methane extraction and spontaneous burning of coal lies in analyzing the coalbed methane extraction process parameters,^11^ studying the spontaneous combustion law of coal bodies surrounding the borehole, minimizing air leakage risks, and implementing measures to improve the extraction efficiency.

Both domestic and international scholars have conducted extensive research on the cohabitation of mine gas with spontaneous coal burning. Wang et al.^12^ and Qiao and Cheng^13^ used a fluid–solid model to analyze the factors of air leakage around roadways and boreholes and clarified that roadway excavation and drilling construction lead to the development of internal cracks in coal bodies. Under negative pressure conditions, a weakened area is formed around the borehole, leading to the formation of air leakage channels. Through simulations and experiment, Li et al.^14^ identified a high-risk area for spontaneous coal combustion caused by air leakage around the borehole. Zheng et al.^15^ and Zhang et al.^16^ studied the multifield evolution of spontaneous coal combustion under the condition of gas in a specific coal mine goaf using a thermal experiment platform and numerical simulation method, revealing the influence relationship of different extraction parameters. Wen et al.^17^ and Zhao et al.^18^ established a seepage model of a goaf and analyzed the variation law of the “three zones” of spontaneous combustion in a goaf during gas extraction. It was concluded that the oxidation risk area of the goaf increased with increasing extraction capacity, which strengthened the oxidation of coal. Zhao et al.^19^ used COMSOL to study the oxidation process of coal around the borehole and analyzed the changes in gas seepage velocity and gas concentration in the extraction borehole. Jia et al.^20^ used a model of the gas flow and temperature field in boreholes and analyzed the influence of extraction pressure and roadway temperature on spontaneous coal combustion around boreholes. Jia et al.^21^ and Xia et al.^22^ used a multifield coupling model of gas extraction in a goaf and discussed the coupling mechanism of spontaneous coal combustion and gas symbiotic disasters in the goaf. It was concluded that an increase in the gas extraction intensity would have a negative impact on the temperature field of a fully mechanized caving goaf, consequently increasing the probability of coal oxidation.

However, the majority of research has used a single parameter and a simple physical field method to study the migration law of gas in the goaf. It is relatively rare to analyze the law of coal oxidation caused by air leakage around the borehole during gas extraction in the original coal seam using the thermal–fluid–solid multiphysical field and multiparameter method.

In summary, based on prior research, to address the issue of predicting and preventing spontaneous coal seam combustion induced by borehole coalbed methane extraction, the coal body is defined as a special porous medium. Using the 215 working faces as an example, based on the thermal–fluid–solid coupling model, we assessed the relationship between air leakage, coal oxidation, and gas desorption and analyzed the coal oxidation mechanism around the borehole. We investigated the effects of the sealing depth, pumping pressure, and pumping time on the oxidation law of coal bodies and established the optimal negative pumping pressure and sealing depth. Lastly, we predicted the boundary line of the most dangerous area for oxidative heating under these parameters. Our results provide a theoretical basis for the prevention of spontaneous combustion of coal bodies around coalbed methane-pumping boreholes and ensuring efficient and safe coalbed methane pumping.

## 2 Mathematical Model Construction

To examine the mathematical model of spontaneous CBM combustion under extraction conditions, the underlying assumptions were as follows:^23^

(1) As an elastic pore fracture medium, free gas and leaking air exist only in the fracture system, and the deformation of the coal is in accordance with the hypothesis of small deformation.
(2) The coal bodies only have an adsorption effect on the gas and have no adsorption properties for other gases. Other gas components of coalbed methane are ignored, and gas is taken as the research object.
(3) Without considering how temperature affects the dynamic viscosity of a gas, the ideal gas state equation is satisfied by air and gas.
(4) The laws governing the migration of substances in the coal body fissures and the air in the pores of coal bodies are described by both Darcy’s law and Fick’s law.
(5) Ignoring the effect of gas in the coal bodies on spontaneous coal combustion, the coal bodies adsorbed oxygen from the roadway to initiate the coal oxidation reaction.

### 2.1 Coal and Rock Deformation Control Equation

Drilling and pumping coal bed methane lowers the gas concentration and pressure, changing the porosity of the coal.^24^ It has been established that the coal seam gas pressure, coal body deformation, and extraction time are related. The bulk of coal-rock-containing gas is thought to be a linearly isotropic material, considering the pore pressure, adsorption expansion stress, and linear elastomer deformation equilibrium equation.^19^
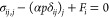
1

The effective stress determined by Hooke’s law determines the magnitude of coal deformation. The displacement and strain components follow the coding ratio. By combining the above equations, the equation related to the coal skeleton displacement and pore pressure is obtained, that is, the controlling equation of coal and rock deformation is^25,26^
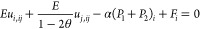
2where δ_*ij*_ is the strain tensor; *F*_*i*_ is the physical force (where *i* = 1, 2, 3, representing *F*_*x*_, *F*_*y*_, and *F*_*z*_, respectively); α is the Biot consolidation coefficient; *p* stands for the gas pressure, where *p* = *p*_1_ + *p*_2_, with *p*_1_ standing for the gas pressure and *p*_2_ standing for the air pressure; *E* is the rock mass’s and coal’s elastic modulus. θ is Poisson’s coal ratio; *u*_*i*,*ij*_ represents the displacement’s second derivative *u*_*i*_ in the direction of *j*; and *u*_*j*,*ij*_ is the derivative of the volume strain *u*_*j*,*i*_ in the direction of *j*.

The ratio of the combined volume of the porous medium to the porous fissure medium determines the porosity of the coal. Considering elements such as coal deformation and gas pressure, the dynamic porosity equation for coal deformation is as follows^27^
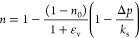
3where *n* represents the coal’s dynamic porosity, *n*_0_ stands for the coal’s initial porosity *n*_0_ = *V*_P0_/*V*_b0_; ε_v_ is the volume strain of coal, ε_v_ = Δ*V*_b_/*V*_b0_; *V*_P0_ and *V*_b0_ are the initial pore volume and initial coal volume, respectively; Δ*V*_b_ is the overall coal volume change; Δ*p* is the gas pressure change; and *k*_s_ stands for the modulus of the skeletal coal.

### 2.2 Control Equation of Gas Seepage in Coal

According to the relationship between the gas density and pressure in the ideal gas state equation combined with the empirical formula of permeability, the governing equations of gas seepage can be obtained.^22,28^
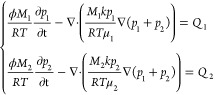
4where *M*_*i*_ represents the molar mass of *i* gas, kg/mol; *R* represents the molar constant for gas, 8.314 J/(mol·k); *T* stands for the coal seam temperature, K; *k* represents the permeability, m^2^; *p*_*i*_ is the *i* gas pressure, MPa; *u*_*i*_ is the kinetic viscosity coefficient of the *i* gas, Pa·s; and *Q*_*i*_ is the source term of the gas, kg/(m^3^·s).

### 2.3 Gas Equation Governing Fissure Coal around the Borehole

During the process of negative pressure gas recovery, the adsorbed gas in the coal and rock diffuses outward, causing the cracks and coal around the borehole to react with oxygen molecules, subsequently triggering a redox reaction that results in the production of CO gas. The governing equation for this reaction is as follows^15^
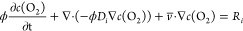
5where *c*(O_2_) is the oxygen concentration, mol/m^3^; *D*_*i*_ stands for the diffusivity of *i* gas, m^2^/s;  is the seepage velocity of the gas mixture within coal, m/s; and *R*_*i*_ is the reactive source term, mol/(m^3^·s), mainly referring to the quality of oxygen consumed per unit time and carbon monoxide produced per unit coal during low-temperature oxidation of fractured coal around the hole. In a previous experimental study, our research group determined the functional relationship between the oxygen consumption rate, gas production rate, heat release intensity, and other characteristic parameters of coal oxidation with the change in temperature and air supply volume, obtained through experimental data fitting, as follows^11^
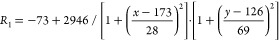
6
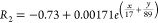
7In the formula, *x* represents the temperature of coal, °C; *y* represents the air supply volume required for coal heating, mL/min; *R*_1_ represents the oxygen consumption source term of the reaction between coal and oxygen; and *R*_2_ represents the source term of the CO gas produced by the reaction of coal with oxygen.

The gas quality control equation is^15^
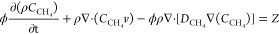
8
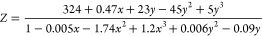
9where *C*_CH_4__ is the mass fraction of gas; ρ is the gas density; and *z* represents the source term of gas desorption.^25^ It is obtained by fitting the relationship between the gas generation rate and the change in the air supply volume.

### 2.4 Control Equation of Heat Transfer of Coal around the Borehole

Heat is released by the oxidation of the coal bodies. The heat transfer follows the energy balance equation, and the control equation is as follows^22^
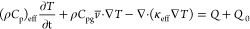
10where  is equivalent to hot melt, J/(m^3^·K); *C*_p_ is the specific heat capacity of the porous media, J/(kg·K); *C*_pg_ is the specific heat capacity, J/(kg·K); κ_eff_ stands for the heat conduction coefficient of gas, J/(m·s·K); and *Q* stands for the heat generated from the coal, J/(m^3^·s). The governing equations of the oxidation reaction heat *Q* and exchange heat *Q*_0_ of fractured coal around the borehole are expressed as
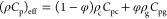
11
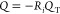
12
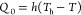
13
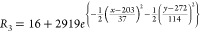
14where *Q*_T_ is the heat generated by the oxidation of the coal-consuming unit molar mass of oxygen (J/mol); *h* is the heat transfer coefficient, W/(m^2^·K); *T*_h_ is the roadway temperature, K; and *R*_3_ represents the source term of the exothermic intensity of the reaction between coal and oxygen.^28^

This equation shows how the coal body surrounding the drilling hole transfers heat.^22^
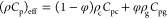
15

The particular heat capacity of porous media is denoted by *C*_p_, J/(kg·K); the equivalent conductivity is κ_eff_ = (1 – φ)^κ_c_ + φκ_g_^, coal and gas have heat transfer coefficients of κ_c_ and κ_g_, respectively, J/mol; and *C*_pc_ represents heat capacity of coal, J/(kg·K).

### 2.5 Thermal–Fluid–Solid Coupling Model

**Figure 1:**
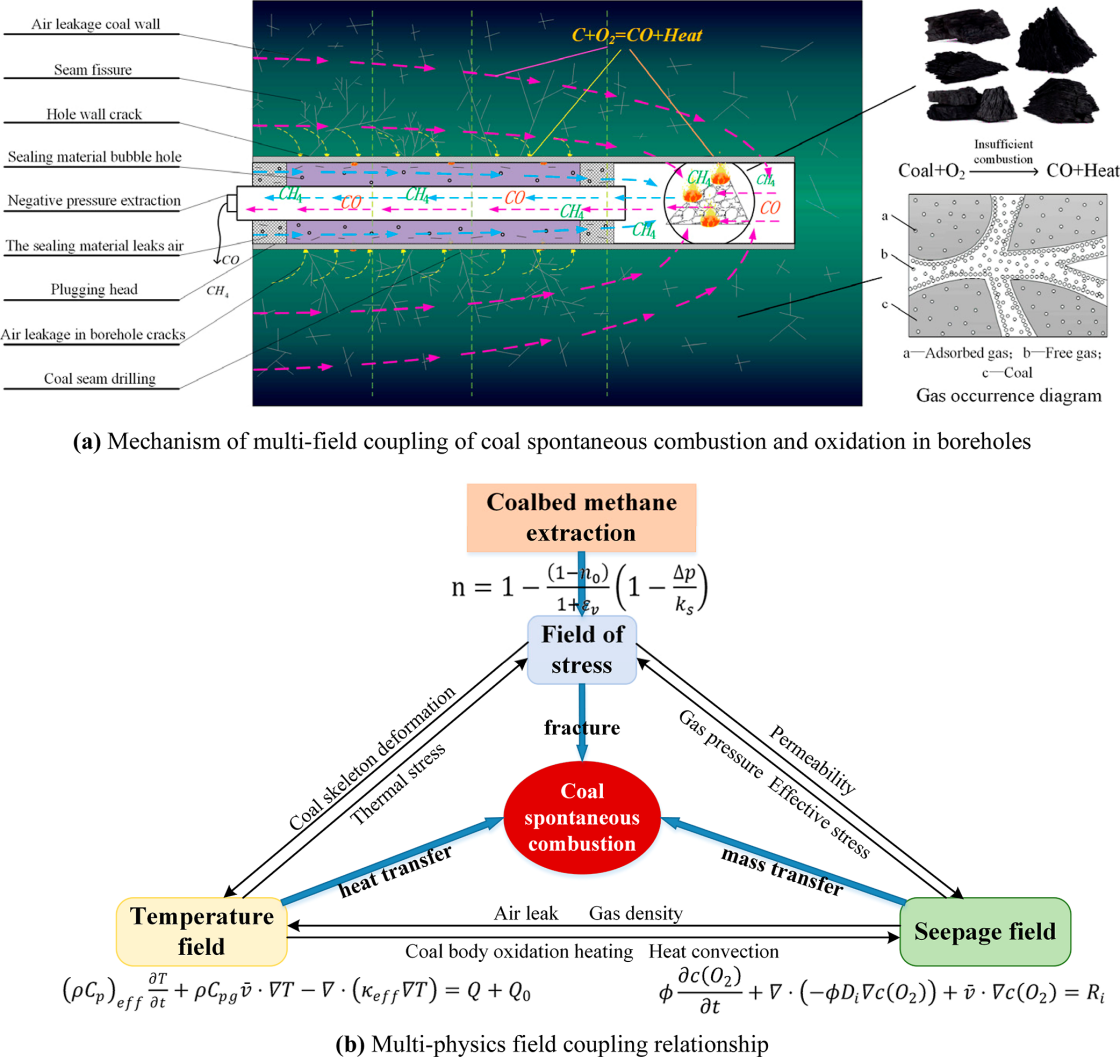
Relationship between thermal, fluid, and solid multifield connection.

Coal seam drilling initiates varying degrees of air leakage under various negative-pressure extraction conditions. With the continuous extraction of negative pressure during drilling, air flows into the coal wall and comes into contact with the drilling hole, forming a closed loop. After drilling, the confining pressure around the borehole is inevitably changed by the activities of the adjacent roadway and coal seam mining, resulting in hole wall collapse, deformation, and diameter shrinkage in the borehole. The broken loose coal and constant oxygen supply in the closed loop are essential for the coal to undergo spontaneous combustion.^19^Figure 1a shows the oxidation mechanism of the coal body surrounding the multifield coupling borehole. The control equation can be solved using COMSOL software to obtain the laws of coal temperature distribution, gas seepage velocity, gas pressure change, and oxygen concentration change.

The above equations were combined to calculate coal porosity *n*, coal oxidation and heat release *Q*, gas flow rate *v*, oxygen concentration *C*, and gas pressure p during gas extraction. The multifield coupling partial differential equations of the thermal–fluid–solid model of gas drainage were established using the equilibrium equation, and the multifield coupling relationship between the stress field, flow field, and temperature field of the gas predrainage borehole was established. Changes in the stress field alter the gas pressure and seepage fields, which in turn affect the coal oxidation temperature and alter the characteristics of the gas pressure and coal seam permeability. The temperature field affects the deformation energy of the stress field and the sorption and desorption of gas in the seepage field, thus altering the flow and gas concentration fields. Finally, the three form a closed-loop multifield coupling, and the spontaneous coal combustion multifield coupling relationship in this case is depicted in Figure 1b.

## 3 Three-Dimensional Model Construction

### 3.1 Test Face Profile

The depth of the coal rake in the coal mine ranged between 520 and 700 m. The working face of this test was 215 units. The mining coal seam had a thickness of 0–12 m, the average inclination of the coal seam was approximately 3°, and the coal body density was 1330 kg/m^3^.

### 3.2 Gas Extraction Geometric Model

**Figure 2:**
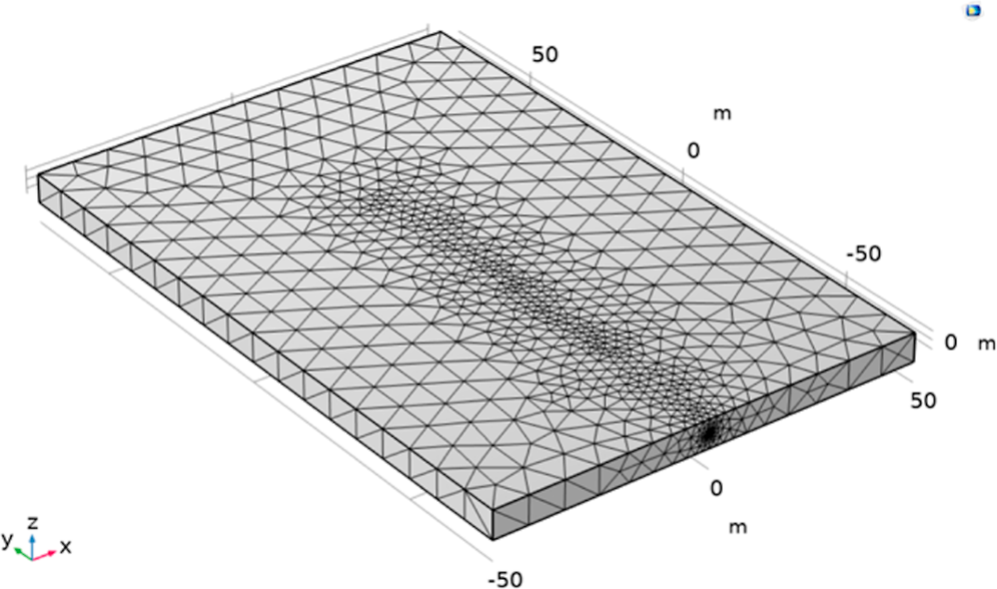
Geometric model and grid analysis of gas extraction boreholes.

A 3D geometric model 100 m in length, 150 m in width, and 6.5 m in height was created using real parameters of the 215 working face as a guide. The actual extraction hole bore was 94 mm, and the deformation and cuttings caused by drilling were taken into account in the modeling process; a borehole with a diameter of 100 mm was positioned in the center of the model, the hole was sealed at a depth of 15 m, the ground stress was 7.5 MPa, and an air leakage channel was installed around the borehole. The established model was divided into grids, and the size of the grid units was refined. The geometric model was divided into 38,471 grid domain units and 4310 boundary units. Figure 2 displays the grid division and computation model.

### 3.3 Boundary Conditions and Model Parameters

The coal seam parameters of 215 belt transportation lanes were used as the basis for the solution. Table 1 lists the parameters used in the numerical simulation.

**Table 1:** Physical Parameters

name of parameter	values and units
elastic modulus of coal	731 MPa
Poisson’s ratio of coal	0.36
initial porosity of coal	7.39%
initial permeability of coal	3.9 × 10^–18^ md
coal density	1330 kg/m^3^
crustal stress	7.5 MPa
dynamic viscosity of gas	1.04 × 10^–5^ Pa·s
dynamic viscosity of air	1.79 × 10^–5^ Pa·s
gas density in typical circumstances	0.717 kg/m^3^
borehole bore	100 mm
negative pressure extraction	18 kPa

The bottom boundary of the coal body is defined as a fixed constraint without displacement. A rod support was placed around the coal body, a certain volume load was applied inside the coal, and the top boundary condition is set as *p*_d_. The specific expressions are as follows^19^
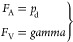
16where *F*_A_ is the boundary load; *F*_*V*_ is the volume load; and  is a variable.

The first boundary condition is selected as the flow field boundary condition. It is set that the coal seam extends infinitely and that the floor and roof are impermeable. The starting temperature of the coal seam was 293 K, and the pressure of the model roadway is designated as *p*_*n*_, as follows^19^
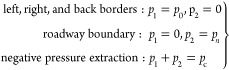
17

### 3.4 Evolution Characteristics of Porosity around the Borehole

**Figure 3:**
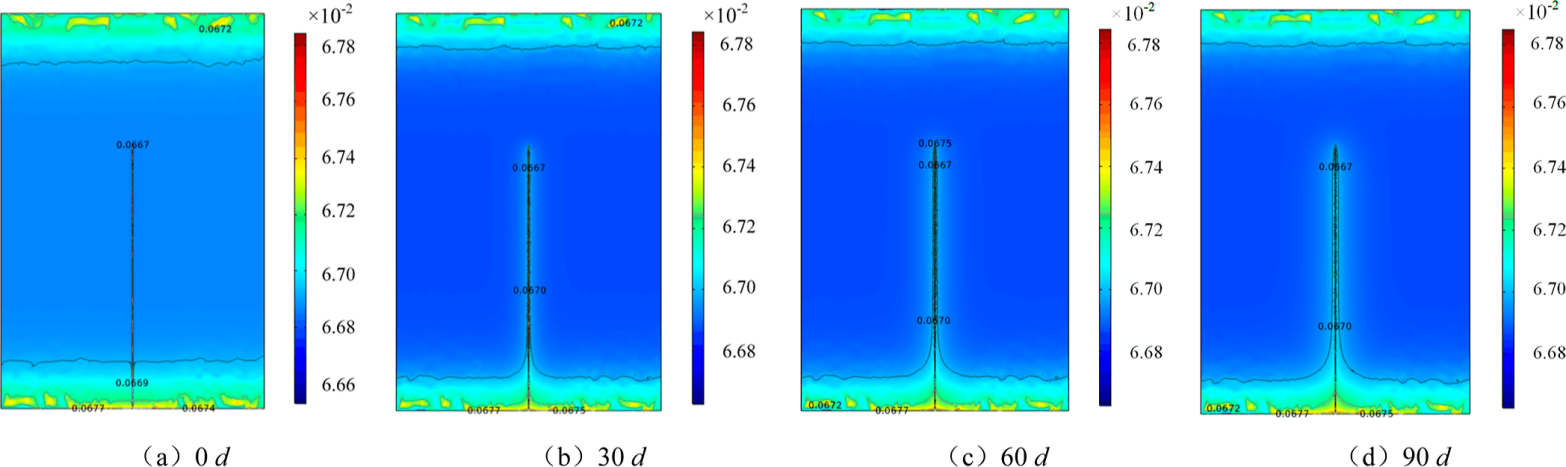
Variation pattern of porosity around the model borehole.

The coal porosity variation law surrounding the gas extraction borehole is shown in Figure 3. The porosity around the borehole presents a symmetrical distribution centered on the extraction borehole. The porosity of the coal rock mass surrounding the drilling hole tends to increase with increasing extraction time due to several factors, such as gas pressure.

### 3.5 Model Validation

Under the 215 working face of the test mine, the drilling ignition was severe, the pumping pressure was 18 kPa, and the sealing depth was 15 m. To further confirm the accuracy of the model, using this working face as the study subjects, 12 new test holes were drilled in the lower side of middle lane 215, among which four holes with a good sealing effect were selected and monitored every 3 days. A CJZ7J(A)/CJZ10J laser gas drainage comprehensive parameter tester (Optics Technology Co., Ltd.) was used to monitor the CO concentration and amount of gas extracted in the borehole, and the results were compared with those of the model.

**Figure 4:**
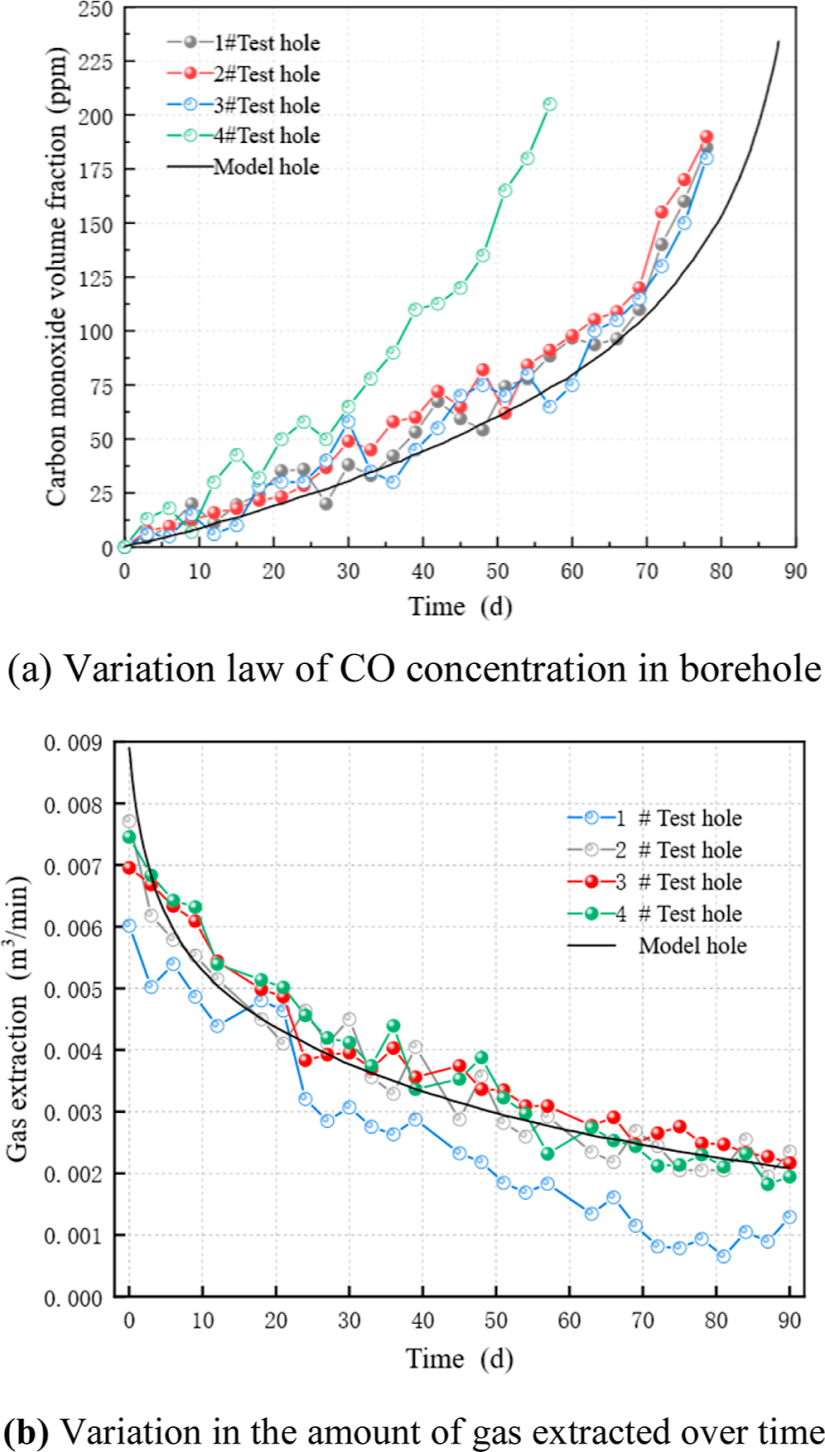
Comparative analysis of field monitoring data and simulation results.

Figure 4a shows a comparison between the gas concentrations monitored on-site and the simulated data. Over time, the CO concentrations in test boreholes 1#, 2#, and 3# gradually increased. On day 80, the concentration increased to 180 ppm. To prevent coal combustion, extraction was stopped. The CO concentration in borehole 4# increased rapidly, reaching more than 200 ppm after 57 d. Among them, the average growth rates of the CO volume fraction in 1#–4# test boreholes are 61.09, 66.22, 59.56, and 78.06 ppm/d, respectively. The change in the CO volume fraction in the monitoring point of the borehole model in the first 70 d was consistent with the CO volume fraction in boreholes 1#, 2#, and 3#. After 70 d, the CO volume fraction in the borehole increased rapidly with time, and the growth rate of the CO volume fraction was 63.74 ppm/d at 90 d. The growth rate differences in CO volume concentration at the monitoring point of the model were 4.18%, 2.65%, and 2.48% in 1#, 2#, and 3#, respectively, and the relative error was small. The growth rate of CO concentration in the model borehole was basically the same as the data obtained from the field test.

Figure 4b shows the g-low–variation curve of the test drilling and the simulation results during the 90 day monitoring period. By comparing the gas extraction amounts of test holes 1#–4# with those of the model holes, it was found that as the time increased, the gas extraction quantities of test holes 2#, 3#, and 4# decreased, and the variation law essentially resembled that of the model holes. The declining trend of gas extraction in the first 30 d of test borehole 1# was similar to that in test boreholes 2#, 3#, and 4#. After 30 d, the declining trend of gas extraction in test borehole 1# was faster, and the gas extraction in the test borehole approached zero when it approached 80 d. Over time, the amount of gas extracted from the model borehole steadily decreased. The amount of gas extracted from the borehole monitoring model within 90 days was 0.0039 m^3^/min. The average gas extraction amounts in 1#–4# were 0.0026, 0.0036, 0.0038, and 0.0038 m^3^/min, respectively. The differences in gas extraction values between the model borehole and the 1#–4# test boreholes are 33.36%, 8.07%, 2.92%, and 3.73%, respectively. As demonstrated by the relative errors of the model boreholes and in boreholes #3 and #4, which are 2.92% and 3.73%, respectively, the numerical simulation model is accurate.

## 4 Results

### 4.1 Impact of Negative Pumping Pressure on Spontaneous Combustion of Coal

The oxygen concentration directly reflects the leakage and combustion states of the coal bodies. The real belt transportation lane situation at the 215 operating face was based on the established thermal–fluid–solid multifield coupling model and numerical simulation. The depth of sealing was 15 m, and the negative extraction pressure was set at 13, 18, 23, and 30 kPa to analyze its influence on the oxygen volume fraction and the coal temperature field surrounding the borehole.

#### 4.1.1 Impact of Negative Pressure Pumping on O_2_ Volume Percentage surrounding a Borehole

**Figure 5:**
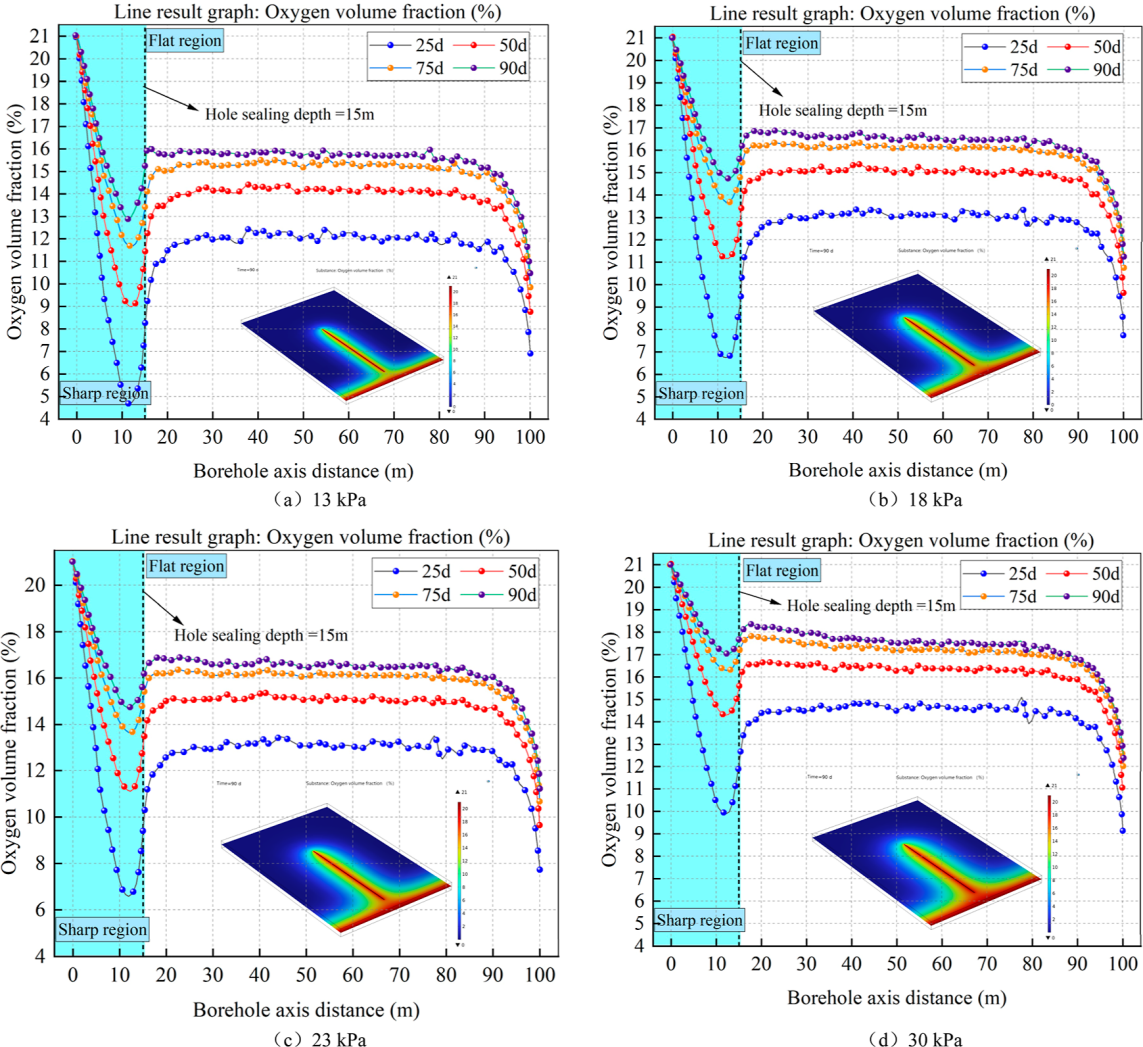
Changes in axial oxygen volume percentage and distribution of oxygen in coal under various negative pressure mining circumstances.

Different negative extraction pressures were set to draw a distribution cloud map of the oxygen fraction around the borehole at 90 days of extraction, and monitoring points were set in the axial direction around the borehole. A three-dimensional axial intercept line 100 m was set at 0.9 m from the borehole wall (the two points of the intercept line were 1,–75,0 and 1,25,0). We plotted the variation curve of the oxygen volume fraction around the borehole with extraction time under different negative extraction pressures within 90 d and obtained the distribution rule of the oxygen volume fraction of the coal bodies under different negative extraction pressures (Figure 5).

The oxygen volume fraction cloud map shows that the coal-filled borehole wall and roadway have the highest oxygen concentrations. The air entering the coal wall during mining causes it to be disturbed, resulting in the formation of large cracks. The coal–oxygen reaction occurs when the roadway air enters the crushing zone of the coal wall, resulting in a gradual decrease in the oxygen content in the axial coal wall along the drilling direction. Similarly, the excavation of the borehole breaks the coal surrounding it, and the coal–oxygen reaction between the air and coal surrounding the borehole under the influence of pressure drainage causes the oxygen content in the borehole center to progressively drop along the radial direction. Overall, as the negative pumping pressure increases, the range of oxygen concentrations around the borehole and in the coal wall of the roadway gradually increases.

From the change curve of the oxygen volume fraction over 90 days, it can be seen that in the axial direction of the borehole, the oxygen volume fraction first rapidly decreased and then gradually increased before stabilizing. A rapid decrease in the oxygen volume fraction occurs because the coal wall is affected by the excavation of the roadway, producing a large fracture zone. Under the action of pressure drainage, air enters the coal wall and oxidizes with the fractured coal bodies. The axial distance of the coal–oxygen consumption rate around the drilling hole increases significantly in the range 0–13 m. The percentage of oxygen at a sealing depth of 15 m will progressively increase due to the high percolation velocity of the side borehole in the sealing section, and it will essentially remain unaltered after an axial distance of 20 m. As observed from the analysis in Figure 5, the oxygen concentration first declined quickly, then gradually increased, and finally stabilized. The oxygen concentration increased progressively as the pumping pressure increased.

#### 4.1.2 Impact of Negative Pumping Pressure on Temperature of Coal surrounding a Borehole

**Figure 6:**
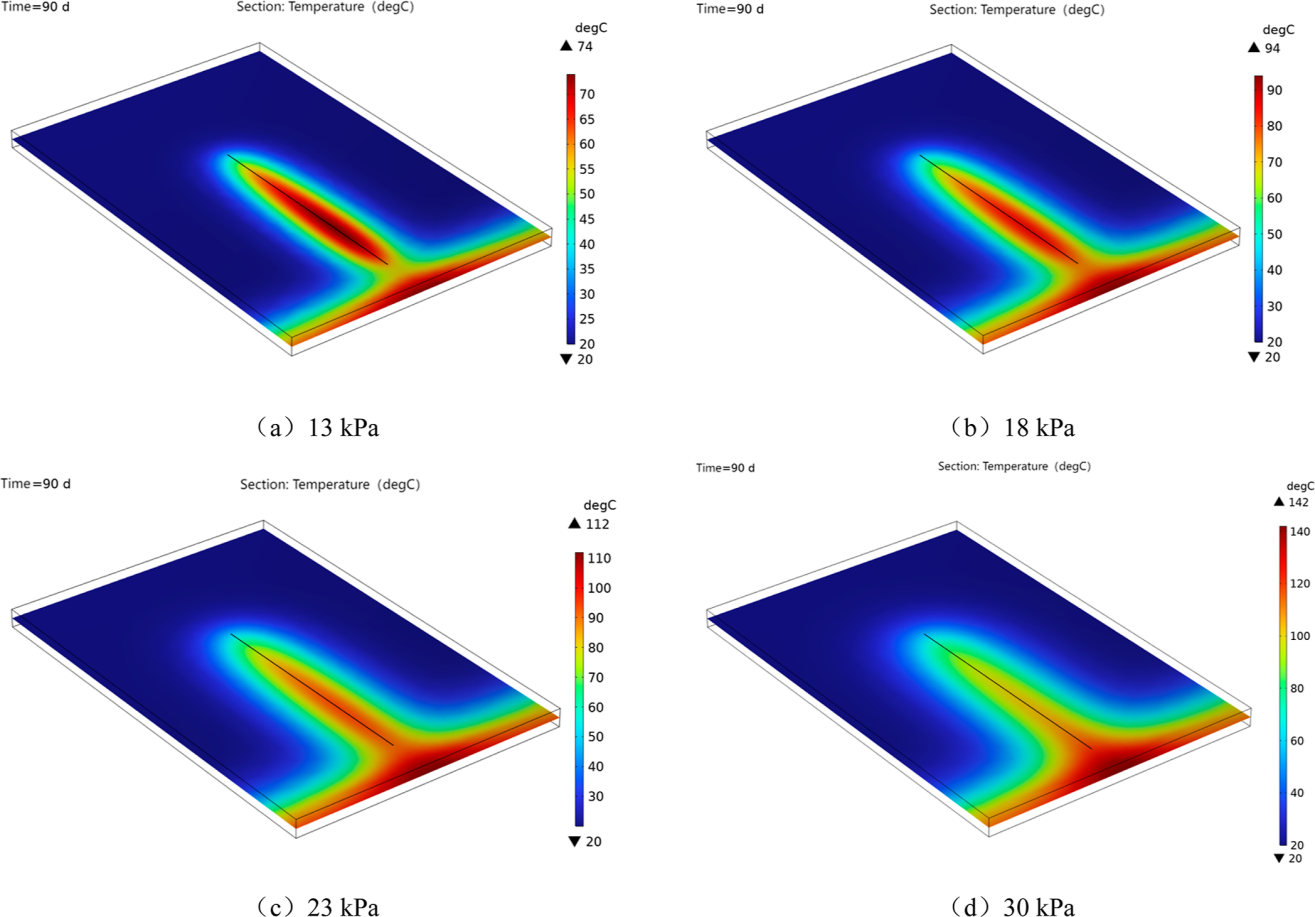
Cloud chart of coal body temperature distribution under different negative pressure conditions.

The coal body temperature distribution cloud map surrounding the gas extraction borehole for 90 days under various negative extraction pressure conditions is shown in Figure 6. The figure illustrates how the temperature inside the coal body increases owing to an increase in negative pressure. At 13, 18, 23, and 30 kPa, the coal body surrounding the drilling hole has a maximum temperature of 74, 94, 112, and 142 °C, respectively.

**Figure 7:**
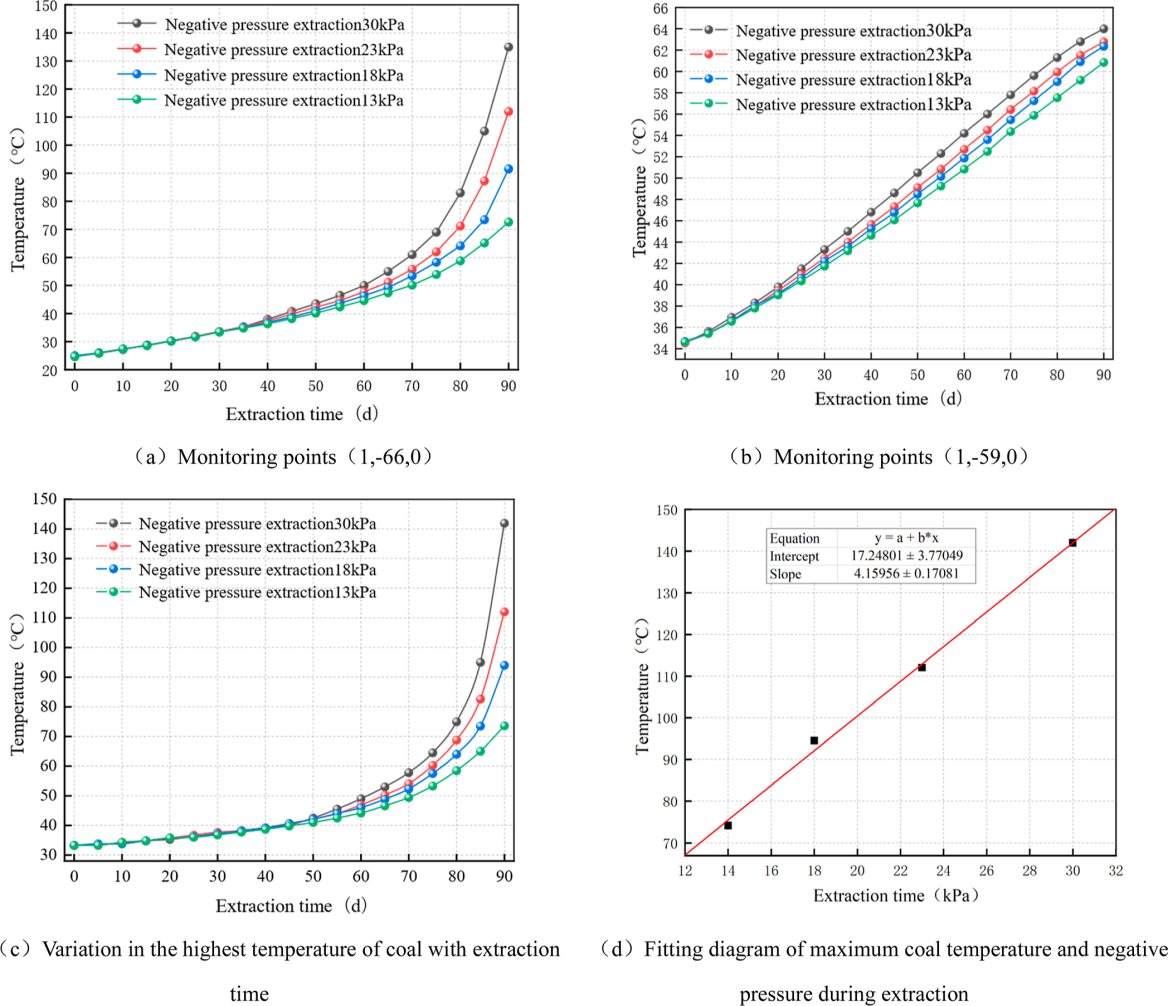
Coal temperature change relationship diagram.

Figure 7a,b shows the variation curves of coal temperature at two monitoring points (1,–66,0) and (1,–59,0) set at the sealing point of the borehole under different extraction negative pressures. The figure shows that as the extraction time increased, the heat accumulated by the coal oxidation reaction increased and the temperature gradually increased. The 40 day extraction period was a node, and the trend of increasing the coal temperature was almost continuous prior to this. Subsequently, it displays varying rates of increase based on the various amounts of oxygen used by various extraction negative pressures. The greatest amount of oxygen consumption occurs at a gas extraction pressure of 30 kPa, which increases the coal temperature to its peak of 135 °C. In contrast, the minimum temperature drops to 73 °C at 13 kPa. Generally, the temperature of the coal surrounding a borehole increases with an increasing negative pumping pressure.

The variation pattern of the highest coal temperature over time under different extraction conditions was plotted according to the temperature distribution cloud map, as shown in Figure 7c. According to the analysis, the temperature increases steadily over time to reach critical temperatures at 88, 83, 81, and 77 days when the negative pressure of extraction is 13, 18, 23, and 30 kPa, respectively. When the extraction time was 90 d, the maximum temperature around the extraction borehole was fitted with the extraction negative pressure, and Figure 7d is obtained. The fitting relationship is as follows
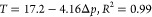
18

This indicates a linear relationship between the highest coal temperature and extraction pressure in the vicinity of the borehole. In summary, based on the temperature field surrounding the extraction drilling hole, it is evident that given the same sealing depth, the coal surrounding the drilling hole is less likely to spontaneously ignite at lower extraction negative pressures.

### 4.2 Impact of Sealing Depth on Spontaneous Combustion of Coal

Inadequate sealing of the borehole caused air to flow into the borehole. When the depth of the sealing hole exceeds the depth value of the stress concentration peak around the roadway, blind spots are created that are not conducive to extraction. A negative pumping pressure of 18 kPa was selected, and the hole sealing depths were set to 15, 18, 21, and 25 m. The influence of the oxygen volume fraction and temperature field variation law on spontaneous coal combustion under different hole sealing depths was analyzed.

#### 4.2.1 Effect of Sealing Depth on O_2_ Volume Fraction around the Drilling Hole

**Figure 8:**
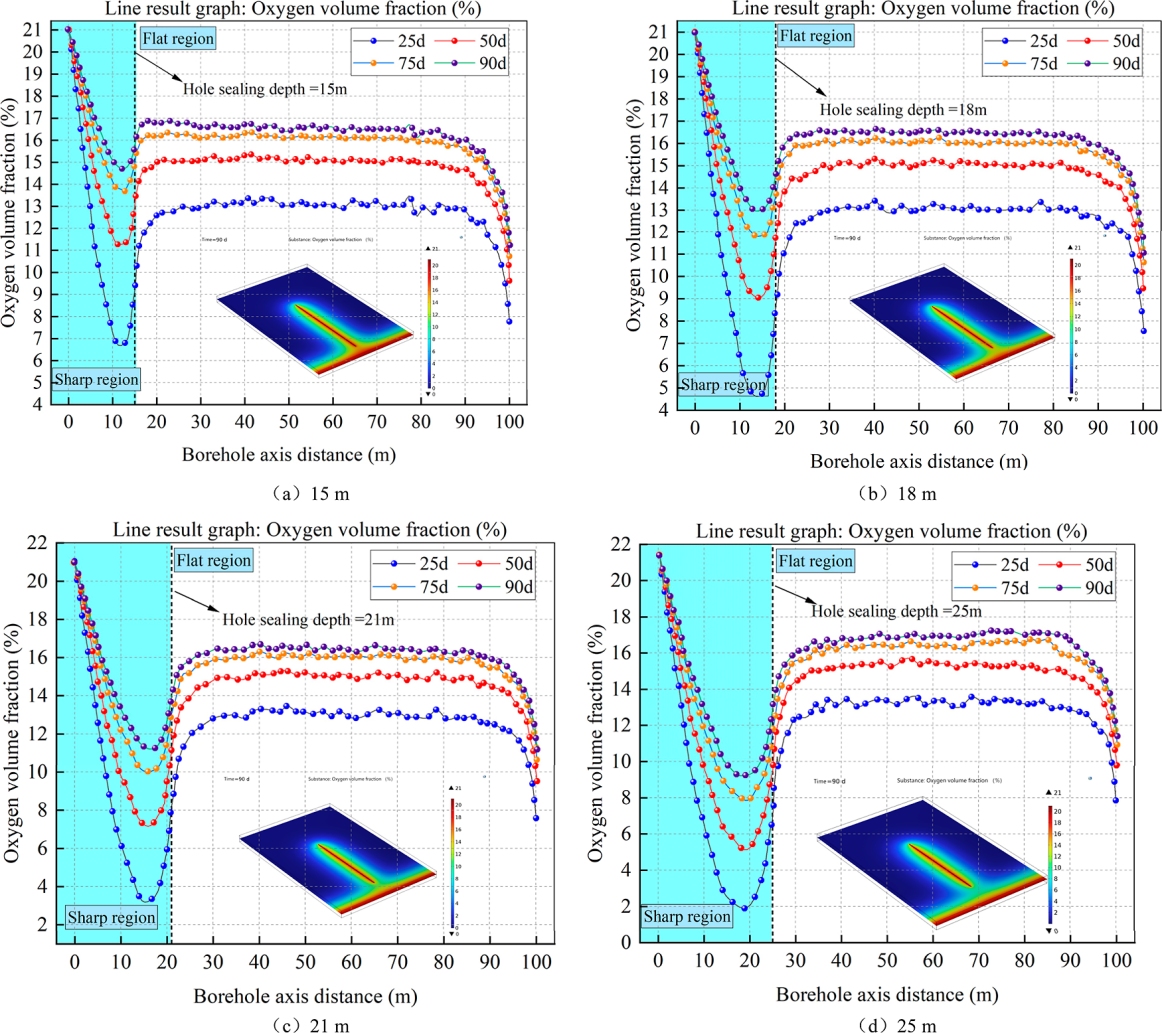
Distribution of the oxygen volume fraction and axial oxygen volume fraction variation in coal under different sealing depths.

Different sealing depths were set, and a distribution cloud diagram of the oxygen concentration around the borehole was drawn after 90 days of extraction. According to the monitoring points arranged in the axial direction around the borehole, the variation curve of the oxygen concentration with extraction time under different sealing depths within 90 days was drawn. The variation rule of the oxygen volume fraction around the borehole at different sealing depths is shown in Figure 8. The oxygen volume fraction cloud map shows that as the hole sealing depth increases, the oxygen volume percentage gradually decreases on the drilling axis; however, it remains virtually constant at the coal wall of the roadway.

From the curve of the oxygen volume fraction change within 90 days, it was observed that in the axial direction of the borehole, the oxygen volume fraction first decreased rapidly, then gradually increased, and then tended to be stable. This is similar to the negative pressure observed in the above analysis. The lowest oxygen volume fraction at the same hole depth at different extraction times was located near the hole sealing depth, but it did not exceed the hole sealing depth. The highest rates of oxygen consumption, the strongest coal–oxygen reactions, and the highest coal body temperatures were observed in this region. Based on a comprehensive analysis, it is evident that as the sealing depth increases, the coal body gradually loses its oxygen volume fraction. Additionally, the highest-temperature area of the coal body gradually shifts from the wall, thereby reducing its impact on the temperature of the tunnel.

#### 4.2.2 Effect of Sealing Depth on Temperature of Coal around the Borehole

**Figure 9:**
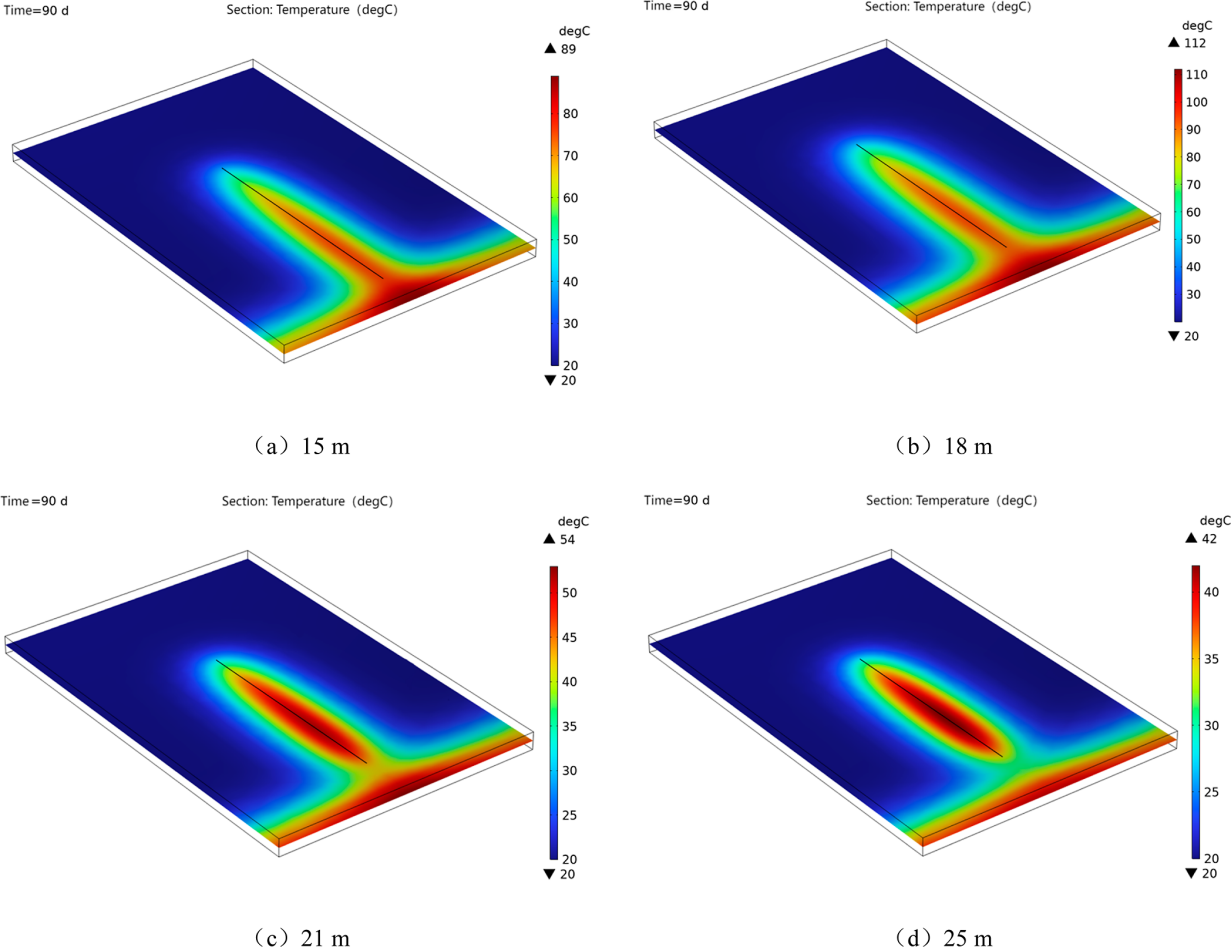
Cloud chart of coal body temperature distribution under different sealing depth conditions.

A distribution cloud map of the coal body temperature when extracting gas from a borehole for 90 days at varying sealing depths is illustrated in Figure 9. The temperature cloud map shows that the fissure coal body surrounding the gas extraction borehole has a maximum temperature of 89, 78, 52, and 38 °C, respectively, depending on the sealing depth of the borehole at 15, 18, 21, and 25 m. The sealing depth controls the variation in the coal temperature surrounding the drilling hole. As the sealing depth increases, the temperature of the coal surrounding the drilling hole decreases. During the excavation operation, the coal wall of the roadway was disturbed, forming a crushing zone, and the voids in the coal body became larger. During pumping, air is directed along the coal wall and into the borehole, where oxygen is continuously supplied to the surrounding coal body, causing it to oxidize.

**Figure 10:**
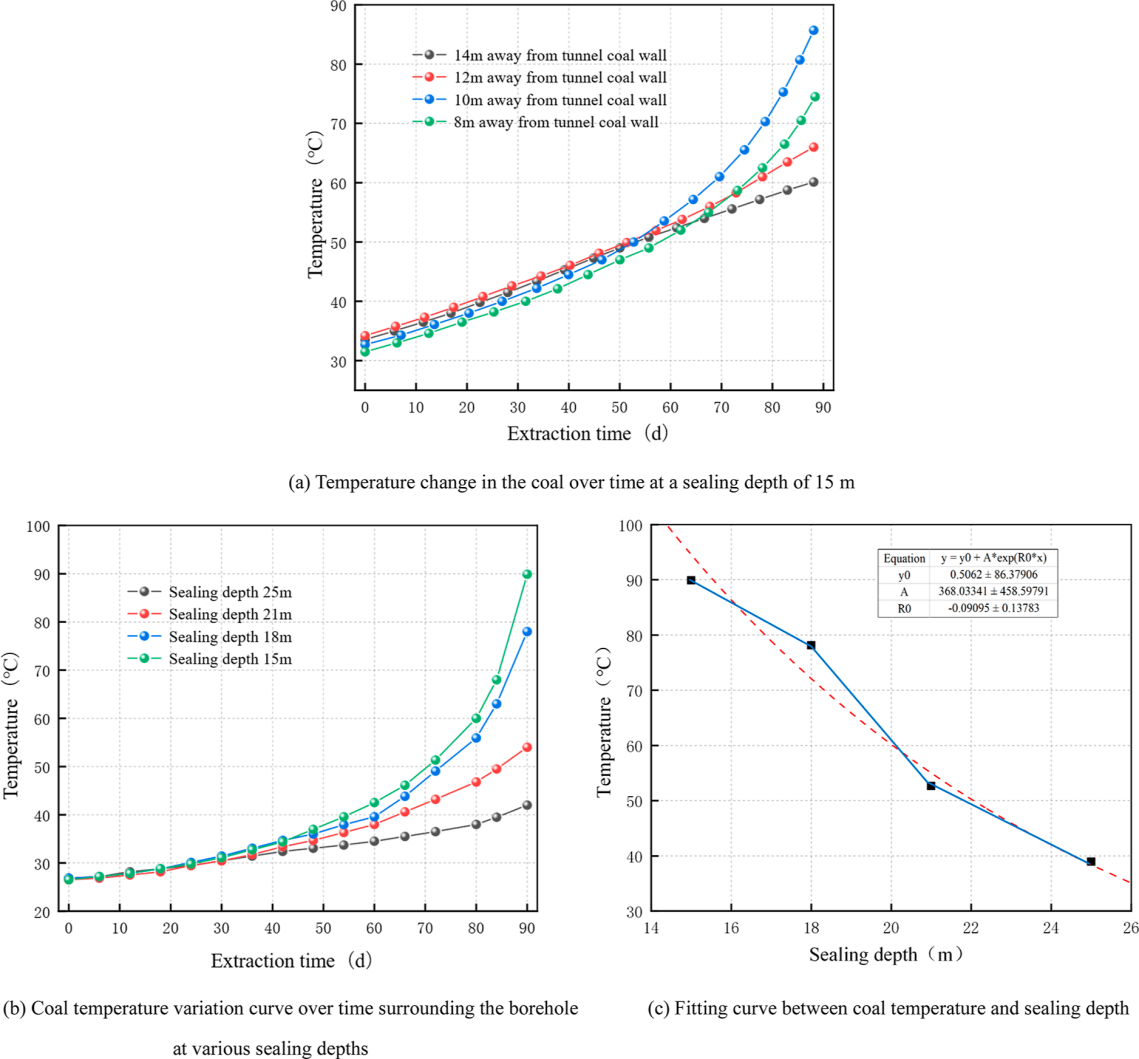
Temperature change in the coal body surrounding boreholes at varying sealing depths.

Taking the model borehole sealing depth of 15 m as an example, four monitoring points were set at positions 8–14 m apart from the coal wall of the roadway to monitor and analyze the temperature field of the coal body. The results are shown in Figure 10a. When the sealing depth was the same, the temperature in the hole gradually increased with time and the rates of change in the monitoring points in the axial direction were different. The coal body temperature near the sealing section of the observation spot 8 m from the coal wall experienced the most rapid increase, whereas the monitoring point at 14 m from the coal wall showed the slowest increase in temperature. This is because air infiltration occurs in the sealing section of the drilling hole, resulting in the oxidation of the surrounding coal bed and subsequent heat release. The coal bodies react more rapidly as the temperature increases. The region creates a hotspot, and heat is transferred by air in the axial direction, increasing the temperature at this location relative to other areas.

Figure 10b shows the variation rule curve of the highest coal temperature over time at different sealing depths plotted based on the cloud map. In the extraction borehole, the greatest temperature of the fractured coal body was observed to be in a positive relation with the extraction time. With increasing sealing depth, the temperature increase rate of the coal body surrounding the drilling hole progressively decreased, and after the critical temperature, the rate of the increase rapidly increased. The experimental results show that the coal body will oxidize quickly after the temperature reaches 70 °C. When the sealing depth is 15 and 18 m, it takes 88 and 86 days, respectively, for the coal body temperature surrounding the drilling hole to reach 70 °C. When the sealing depth is between 21 and 25 m, the coal temperature does not reach the critical temperature until 90 days of extraction. This indicates that the temperature increase pattern is essentially the same for sealing depths of 15 and 18 m. The coal body surrounding the borehole does not oxidize or increase the coal temperature too quickly if the sealing depth is less than 18 m. When the time was 90 d, the highest temperature and the sealing depth were fitted and compared with the actual sealing depth (Figure 10c). There was a negative correlation between the sealing depth and temperature of the coal, and the fitting relation is
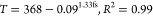
19

By comparing and analyzing the actual sealing depth, we observed that when the borehole depth is 15–18 m, the coal temperature decreases slowly. When the borehole depth is 18–21 m, the coal temperature decreases rapidly. When the temperature of the coal is less than the critical temperature of 70 °C, the borehole depth should be greater than 18 m.

### 4.3 Influence of Extraction Parameters on the Oxidation Heating Zone

**Figure 11:**
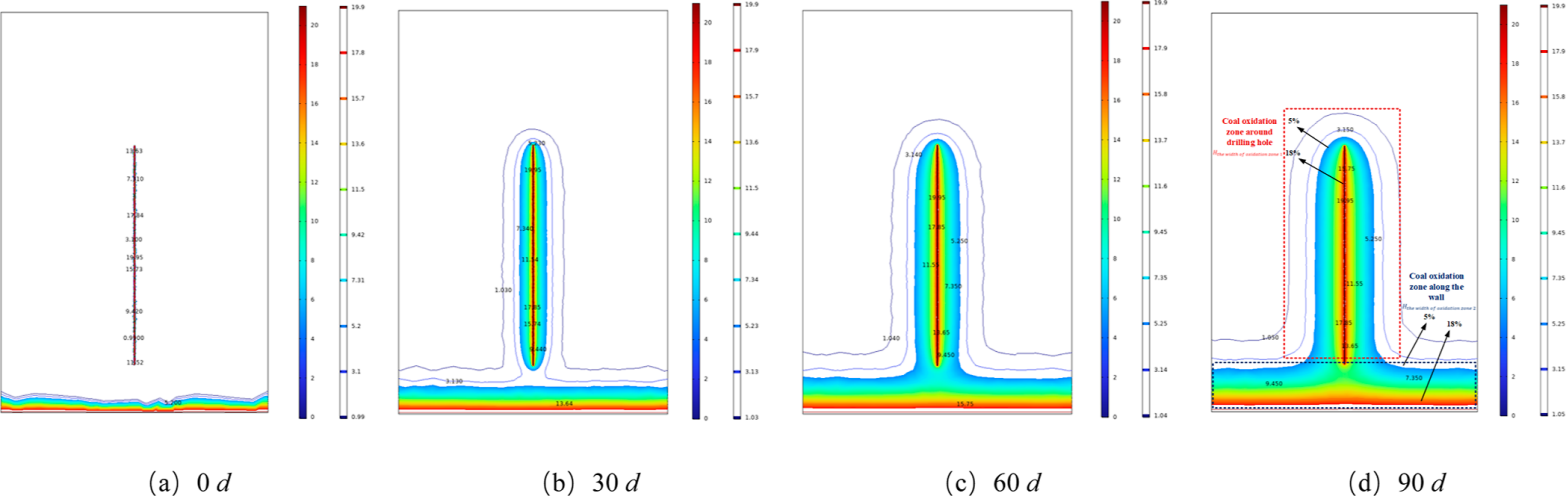
Variation law of oxidation temperature increase area.

Referring to the division standard of oxidation “three zones” of loose coal in the goaf, the oxidation zone of coal around the gas predrainage borehole is divided based on the boundary threshold range of 5% and 18%.^17^ Furthermore, the development law regulating the oxidation zone boundary over time is analyzed. Figure 11 represents the variation curve of the oxidation zone area with time under the condition of negative pressure of 13 kPa and sealing depth of 18 m. As shown in Figure 11d, the oxidation zone in the figure is divided into two parts: the coal oxidation zone along the wall direction *H*_the width of oxidation zone 1_ and the coal oxidation zone around the borehole *H*_the width of oxidation zone 2_. An analysis of this law shows that the oxidation zone area is symmetrically distributed around the extraction borehole. With an increase in extraction time, the oxidation zone of the coal body gradually shifts from the wall direction toward the deeper part of the coal body along the radial direction of the borehole. The oxidation zone of the coal body around the borehole extends along the radial direction of the borehole, and the dangerous area of oxidation and heating increases gradually.

**Figure 12:**
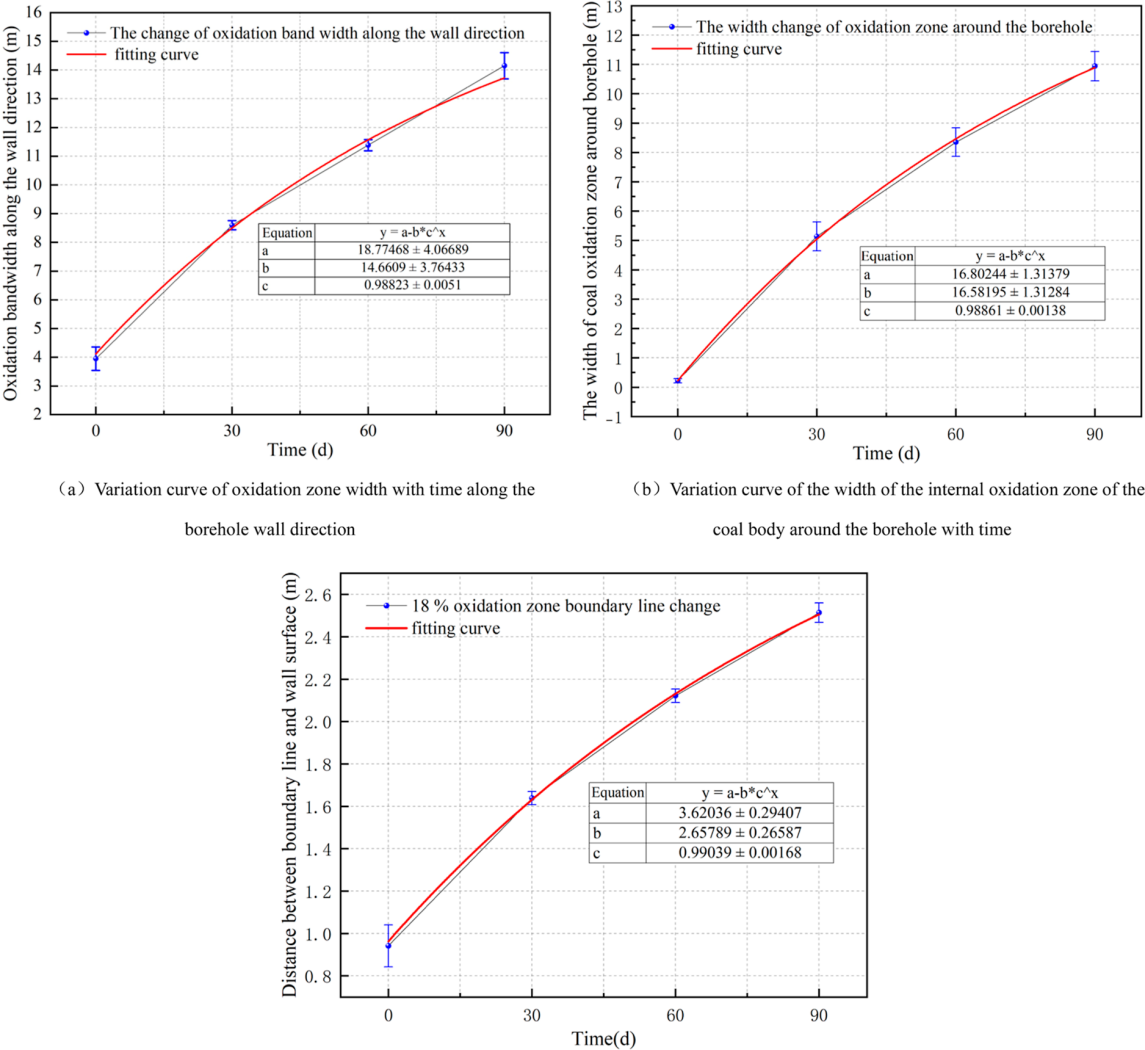
Fitting curve of the oxidation heating area changing with extraction time.

In order to further determine the location characteristics of the oxidation zone, the coal oxidation zone area along the wall direction and the coal oxidation zone area around the borehole are studied. Two axial intercept monitoring points 20 and 40 m from the borehole were set on both sides of the borehole, and three radial intercepts were set at the center of the borehole and 20 m from the center of the borehole. The depths of the boundary line of 5% and 18% oxygen concentrations on the four axial intercepts and three radial intercepts within 90 d of extraction were obtained. The width of the oxidation zone along the wall direction and the width of the oxidation zone around the borehole can be obtained by calculating the difference. The oxidation zone of coal around the borehole takes the borehole as the boundary line; therefore, analysis of the width of the oxidation zone can predict its position. For the oxidation along the wall direction, the 18% oxygen concentration line needs to be used as its boundary line to predict the position. The relationship between the width of the oxidation zone and the position of the 18% boundary line of the oxygen concentration within 90 d of extraction was fitted with the extraction time, and the fitting curve is shown in Figure 12a–c. The diagram indicates a positive correlation between the oxygen concentration boundary line at 18% and the width of the oxidation heating zone, with respect to the extraction time. The fitting relationship could predict the range of the coal oxidation zone and the specific location of the oxidation zone boundary line. The fitting relationships are as follows
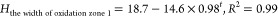
20
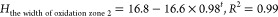
21
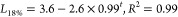
22

## 5 Conclusions

(1) With an increase in negative pressure, the air leakage rate of coal around the borehole sealing section increased. The oxygen consumption rate of the coal increased rapidly to its peak value and then decreased rapidly between the coal wall side and the inner side of the plugging section. The oxygen consumption rate generally increased, which promoted the oxidation of coal. The longer the extraction period is, the hotter the coal is surrounding the borehole, and the maximum coal temperature surrounding the borehole and the negative extraction pressure exhibit a linear relationship, that is, *T* = 17.2 – 4.16Δ*p*, *R*^2^ = 0.99.
(2) The deeper the sealing depth, the smaller the oxygen volume fraction of the coal oxidation reaction; as the extraction time increases, the coal temperature surrounding the plugging part increases. The coal temperature surrounding the drilling hole steadily decreases with increasing sealing depth, and the fitting relation is *T* = 368 – 0.09^1.33fs^, *R*^2^ = 0.99. The sealing depth of the drilling hole should be more than 18 m when the coal temperature surrounding it is below the critical temperature of 70 °C.
(3) Taking into consideration the actual situation of gas extraction at the 215 working face of the coal mine, the optimal extraction parameters are as follows: the extraction negative pressure is 13 kPa and the sealing depth is greater than 18 m, which can effectively prevent the oxidation of coal around the borehole. The relationship between the oxidation zone and time under this parameter was predicted, and the relationship is *H*_the width of oxidation zone 1_ = 18.7 – 14.6 × 0.98^*t*^, *H*_the width of oxidation zone 2_ = 16.8 – 16.6 × 0.98^*t*^, and *L*_18%_ = 3.6 – 2.6 × 0.99^*t*^. The research results provide an important theoretical basis for the prediction of the oxidation range of coal around the predrainage borehole of coalbed methane for the scientific prevention and management of spontaneous combustion of coal around the borehole.
